# Bone chip versus myositis ossificans after supracondylar fracture of humerus

**DOI:** 10.11604/pamj.2022.41.218.31732

**Published:** 2022-03-16

**Authors:** Chanan Goyal, Jyotirmoy Roychowdhury

**Affiliations:** 1 Government Physiotherapy College, Raipur, India,; 2 Datta Meghe Institute of Medical Sciences, Wardha, India,; 3 NH MMI Narayana Superspeciality Hospital, Raipur, India

**Keywords:** Bone chip, myositis ossificans, supracondylar humerus fracture

## Image in medicine

A 44-year-old male presented to the department of physiotherapy with the primary concern of inability to fully straighten his right elbow. As per the history given by the patient, he had sustained a fall on an outstretched hand 10 months ago. He had sustained a fracture around the elbow, which was managed conservatively with plaster cast for six weeks by a local practitioner. He had pain and swelling around the elbow after the injury, but it had subsided two months ago. On examination, terminal 30 degrees of the range of motion (ROM) of right elbow was limited. Although, forearm supination-pronation was full. The X-ray revealed bone chip at the anterior aspect of the distal humerus, along with supracondylar fracture of the humerus that was not completely healed. Bone chip can be differentiated from myositis ossificans, which is a common complication of fractures around the elbow, by its well-defined margins. As a part of physiotherapy intervention, gentle active ROM of right elbow and functional use of right hand for light activities of daily living was emphasized. The patient was advised to avoid use of heat, massage and jerky movements of elbow as these can lead to myositis ossification. He was assured that he had functional ROM of 30 degrees to 130 degrees elbow flexion, so lack of terminal extension should not bother him in his lifestyle.

**Figure 1:**
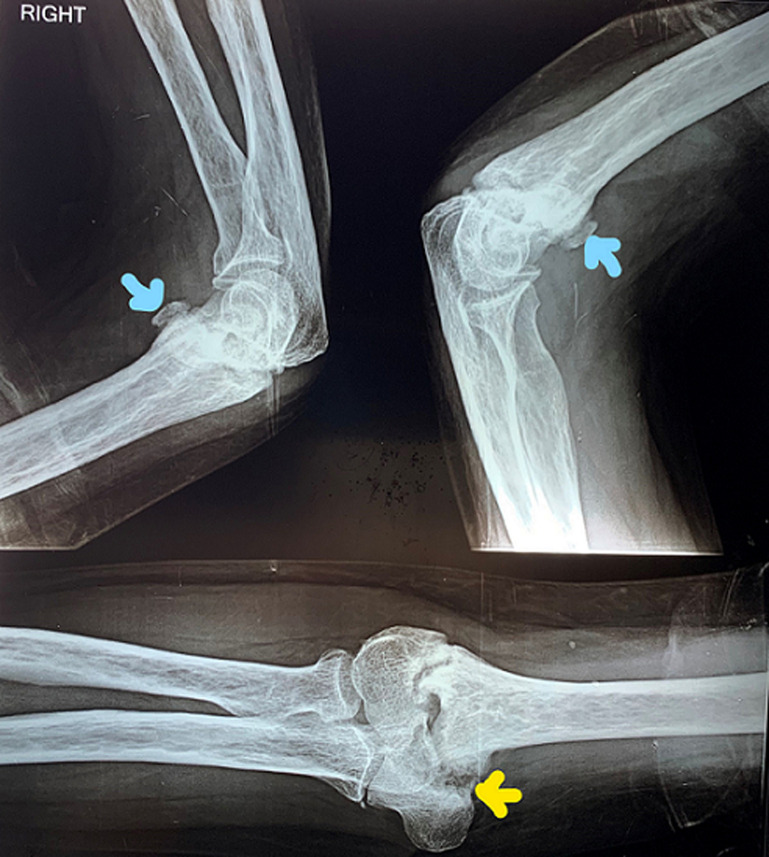
X-ray showing right elbow in lateral and antero-posterior (AP) views (blue arrows show bone chip and yellow arrow shows unhealed supracondylar fracture of humerus)

